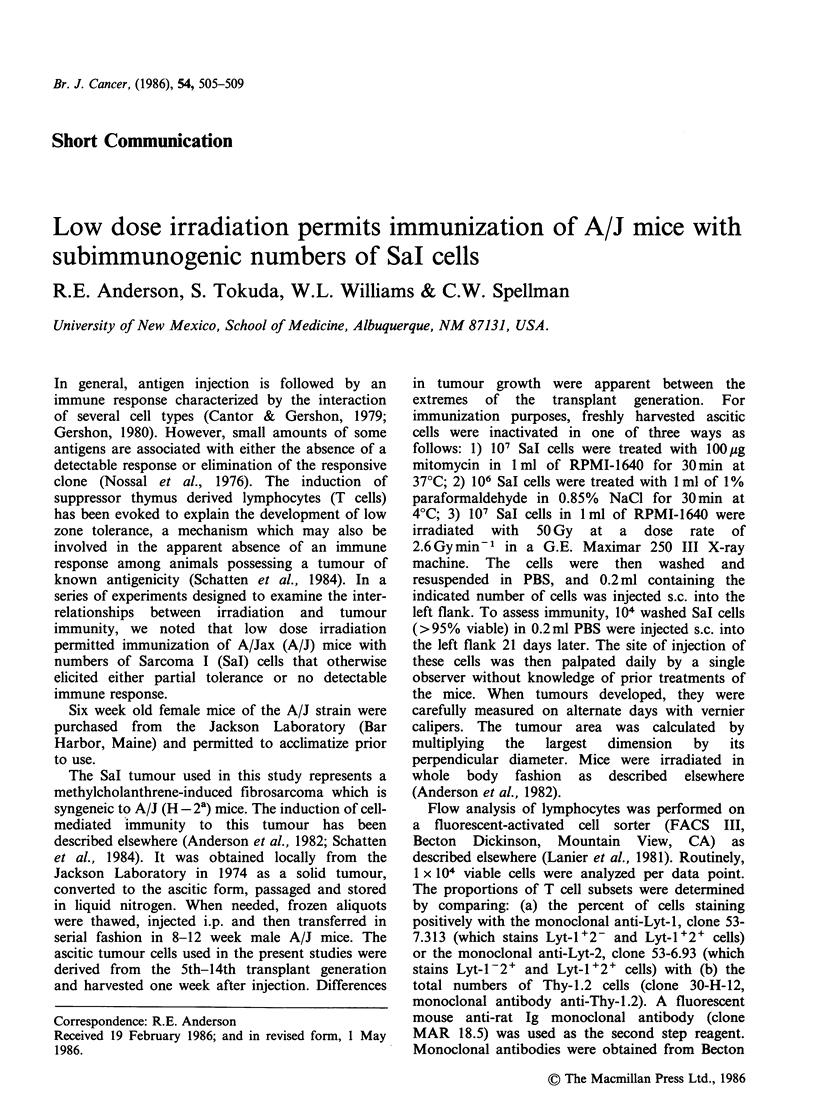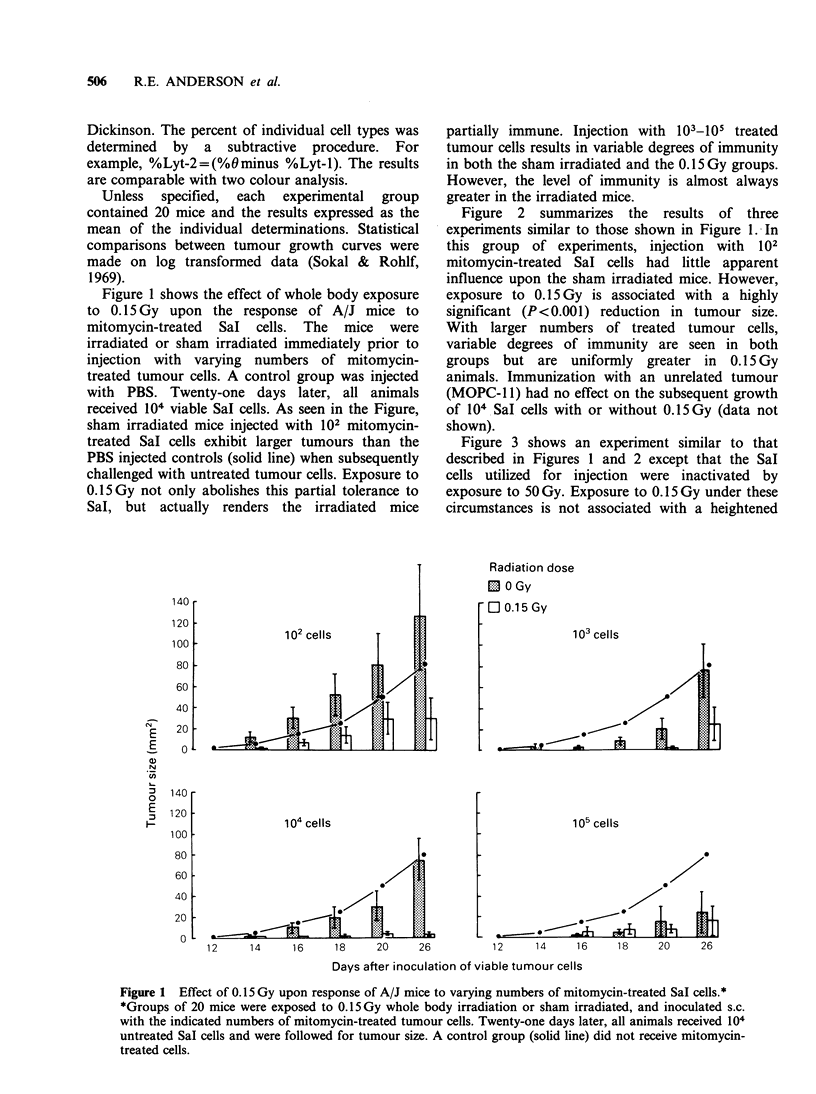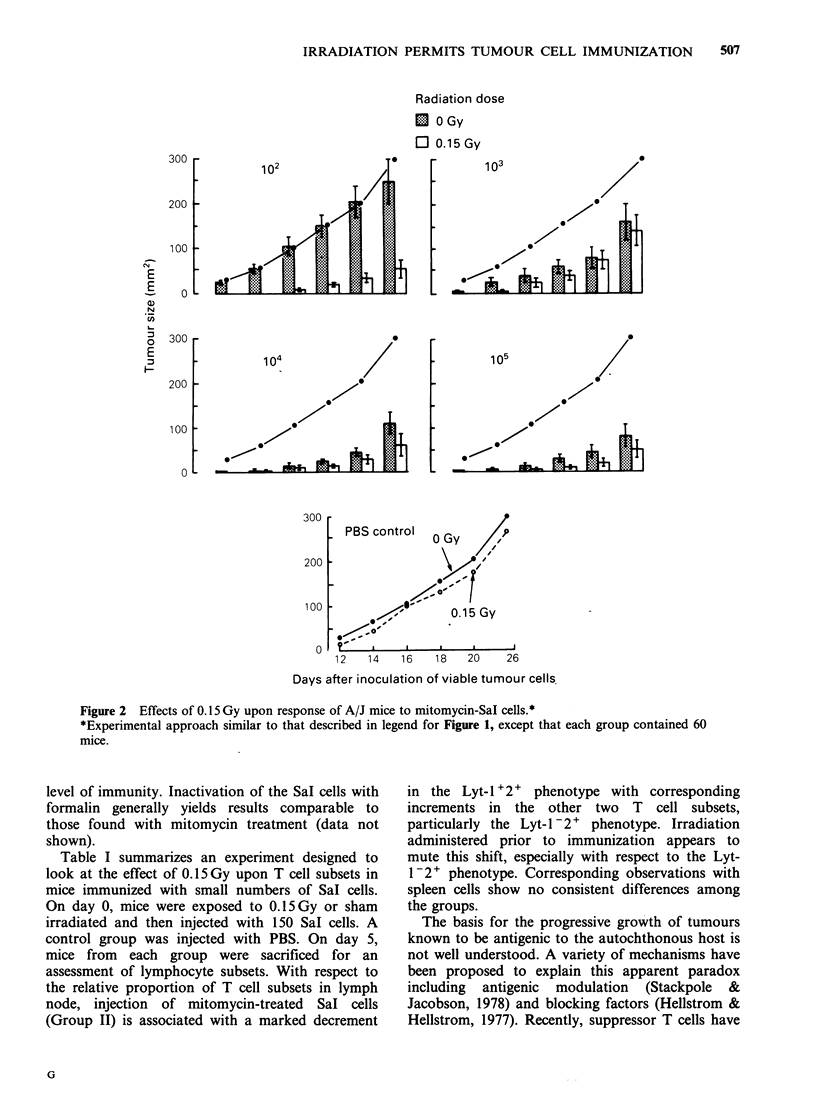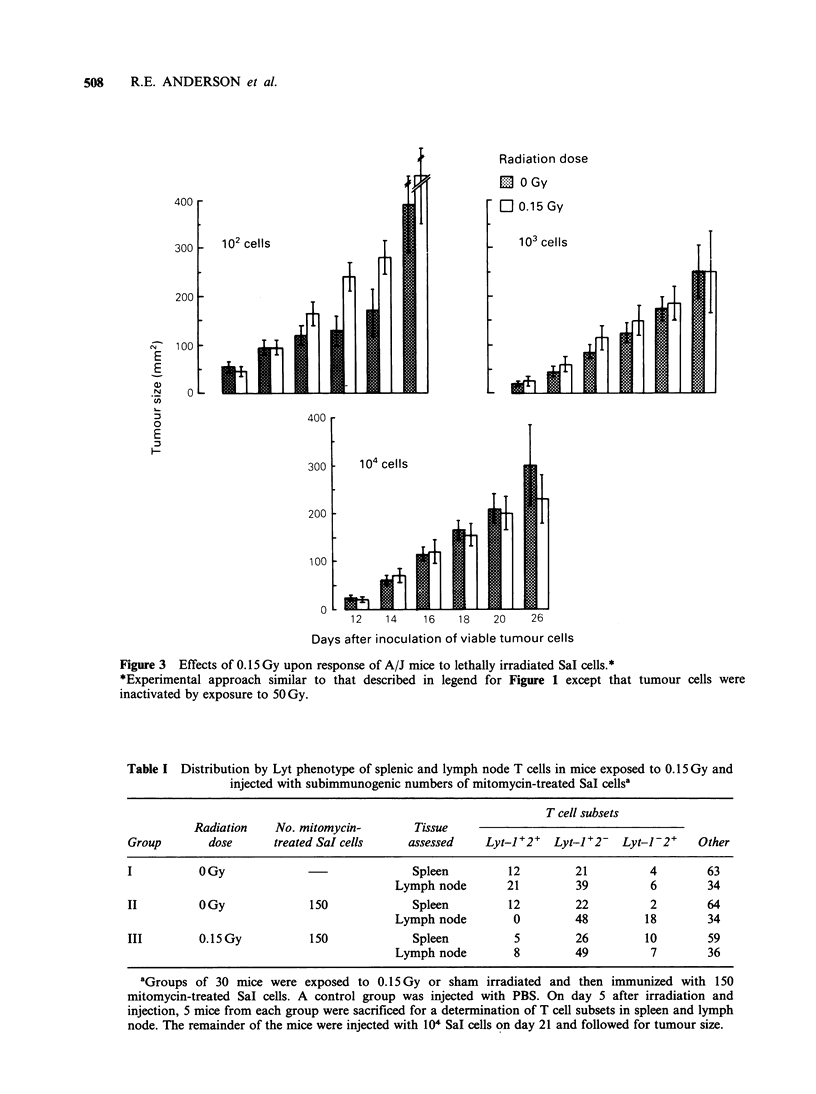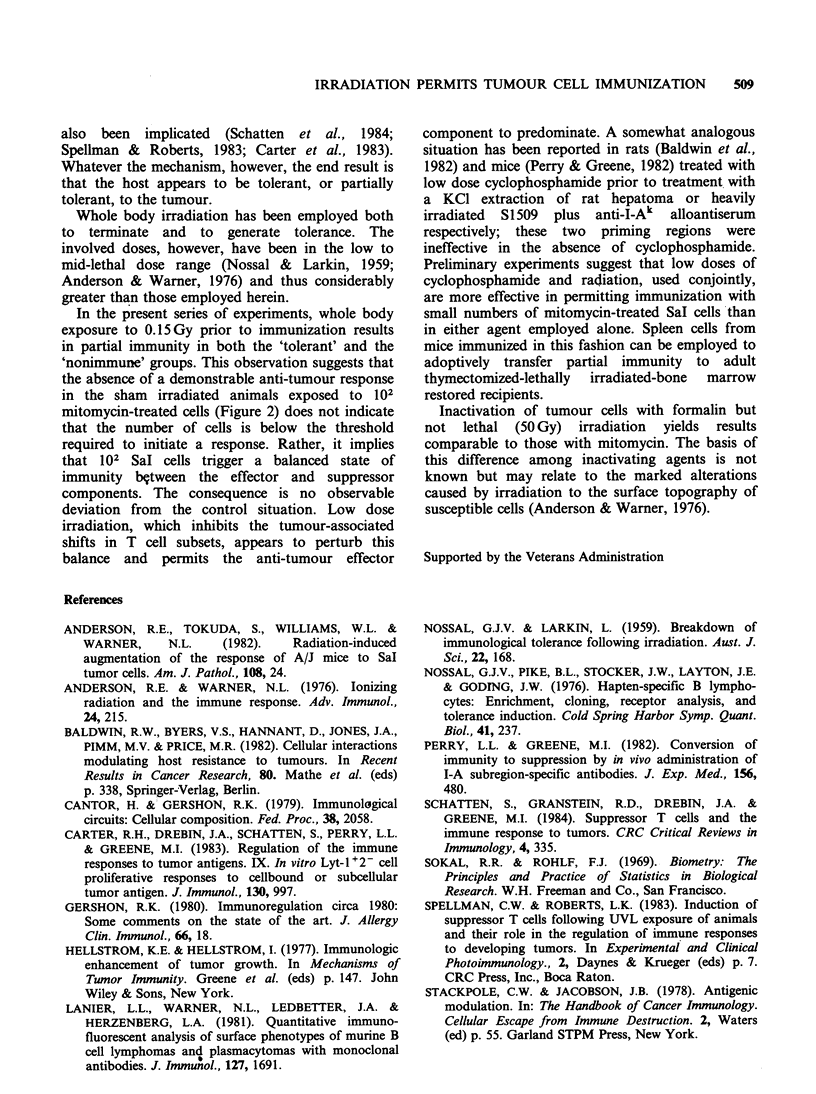# Low dose irradiation permits immunization of A/J mice with subimmunogenic numbers of SaI cells.

**DOI:** 10.1038/bjc.1986.204

**Published:** 1986-09

**Authors:** R. E. Anderson, S. Tokuda, W. L. Williams, C. W. Spellman


*Br. J. Cancer*, (1986), **54**, 505–509

**Short Communication**

### Low dose irradiation permits immunization of A/J mice with subimmunogenic numbers of SaI cells

R.E. Anderson, S. Tokuda, W.L. Williams & C.W. Spellman

*University of New Mexico, School of Medicine, Albuquerque, NM 87131, USA.*

In general, antigen injection is followed by an immune response characterized by the interaction of several cell types (Cantor & Gershon, 1979; Gershon, 1980). However, small amounts of some antigens are associated with either the absence of a detectable response or elimination of the responsive clone (Nossal *et al.*, 1976). The induction of suppressor thymus derived lymphocytes (T cells) has been evoked to explain the development of low zone tolerance, a mechanism which may also be involved in the apparent absence of an immune response among animals possessing a tumour of known antigenicity (Schatten *et al.*, 1984). In a series of experiments designed to examine the interrelationships between irradiation and tumour immunity, we noted that low dose irradiation permitted immunization of A/Jax (A/J) mice with numbers of Sarcoma I (SaI) cells that otherwise elicited either partial tolerance or no detectable immune response.

Six week old female mice of the A/J strain were purchased from the Jackson Laboratory (Bar Harbor, Maine) and permitted to acclimatize prior to use.

The SaI tumour used in this study represents a methylcholanthrene-induced fibrosarcoma which is syngeneic to A/J ($H-2^a$) mice. The induction of cell-mediated immunity to this tumour has been described elsewhere (Anderson *et al.*, 1982; Schatten *et al.*, 1984). It was obtained locally from the Jackson Laboratory in 1974 as a solid tumour, converted to the ascitic form, passaged and stored in liquid nitrogen. When needed, frozen aliquots were thawed, injected i.p. and then transferred in serial fashion in 8–12 week male A/J mice. The ascitic tumour cells used in the present studies were derived from the 5th–14th transplant generation and harvested one week after injection. Differences in tumour growth were apparent between the extremes of the transplant generation. For immunization purposes, freshly harvested ascitic cells were inactivated in one of three ways as follows: 1) $10^7$ SaI cells were treated with 100 $\mu$g mitomycin in 1 ml of RPMI-1640 for 30 min at 37°C; 2) $10^6$ SaI cells were treated with 1 ml of 1% paraformaldehyde in 0.85% NaCl for 30 min at 4°C; 3) $10^7$ SaI cells in 1 ml of RPMI-1640 were irradiated with 50 Gy at a dose rate of 2.6 Gy min$^{-1}$ in a G.E. Maximar 250 III X-ray machine. The cells were then washed and resuspended in PBS, and 0.2 ml containing the indicated number of cells was injected s.c. into the left flank. To assess immunity, $10^4$ washed SaI cells (>95% viable) in 0.2 ml PBS were injected s.c. into the left flank 21 days later. The site of injection of these cells was then palpated daily by a single observer without knowledge of prior treatments of the mice. When tumours developed, they were carefully measured on alternate days with vernier calipers. The tumour area was calculated by multiplying the largest dimension by its perpendicular diameter. Mice were irradiated in whole body fashion as described elsewhere (Anderson *et al.*, 1982).

Flow analysis of lymphocytes was performed on a fluorescent-activated cell sorter (FACS III, Becton Dickinson, Mountain View, CA) as described elsewhere (Lanier *et al.*, 1981). Routinely, $1 \times 10^4$ viable cells were analyzed per data point. The proportions of T cell subsets were determined by comparing: (a) the percent of cells staining positively with the monoclonal anti-Lyt-1, clone 53-7.313 (which stains Lyt-$1^+2^-$ and Lyt-$1^+2^+$ cells) or the monoclonal anti-Lyt-2, clone 53-6.93 (which stains Lyt-$1^-2^+$ and Lyt-$1^+2^+$ cells) with (b) the total numbers of Thy-1.2 cells (clone 30-H-12, monoclonal antibody anti-Thy-1.2). A fluorescent mouse anti-rat Ig monoclonal antibody (clone MAR 18.5) was used as the second step reagent. Monoclonal antibodies were obtained from Becton

Correspondence: R.E. Anderson
Received 19 February 1986; and in revised form, 1 May 1986.

© The Macmillan Press Ltd., 1986

Dickinson. The percent of individual cell types was determined by a subtractive procedure. For example, %Lyt-2 = (%$\theta$ minus %Lyt-1). The results are comparable with two colour analysis.

Unless specified, each experimental group contained 20 mice and the results expressed as the mean of the individual determinations. Statistical comparisons between tumour growth curves were made on log transformed data (Sokal & Rohlf, 1969).

Figure 1 shows the effect of whole body exposure to 0.15 Gy upon the response of A/J mice to mitomycin-treated SaI cells. The mice were irradiated or sham irradiated immediately prior to injection with varying numbers of mitomycin-treated tumour cells. A control group was injected with PBS. Twenty-one days later, all animals received $10^4$ viable SaI cells. As seen in the Figure, sham irradiated mice injected with $10^2$ mitomycin-treated SaI cells exhibit larger tumours than the PBS injected controls (solid line) when subsequently challenged with untreated tumour cells. Exposure to 0.15 Gy not only abolishes this partial tolerance to SaI, but actually renders the irradiated mice partially immune. Injection with $10^3$–$10^5$ treated tumour cells results in variable degrees of immunity in both the sham irradiated and the 0.15 Gy groups. However, the level of immunity is almost always greater in the irradiated mice.

Figure 2 summarizes the results of three experiments similar to those shown in Figure 1. In this group of experiments, injection with $10^2$ mitomycin-treated SaI cells had little apparent influence upon the sham irradiated mice. However, exposure to 0.15 Gy is associated with a highly significant ($P < 0.001$) reduction in tumour size. With larger numbers of treated tumour cells, variable degrees of immunity are seen in both groups but are uniformly greater in 0.15 Gy animals. Immunization with an unrelated tumour (MOPC-11) had no effect on the subsequent growth of $10^4$ SaI cells with or without 0.15 Gy (data not shown).

Figure 3 shows an experiment similar to that described in Figures 1 and 2 except that the SaI cells utilized for injection were inactivated by exposure to 50 Gy. Exposure to 0.15 Gy under these circumstances is not associated with a heightened

**Figure 1** Effect of 0.15 Gy upon response of A/J mice to varying numbers of mitomycin-treated SaI cells.* *Groups of 20 mice were exposed to 0.15 Gy whole body irradiation or sham irradiated, and inoculated s.c. with the indicated numbers of mitomycin-treated tumour cells. Twenty-one days later, all animals received $10^4$ untreated SaI cells and were followed for tumour size. A control group (solid line) did not receive mitomycin-treated cells.

**Figure 2** Effects of 0.15 Gy upon response of A/J mice to mitomycin-SaI cells.*
*Experimental approach similar to that described in legend for **Figure 1,** except that each group contained 60 mice.

level of immunity. Inactivation of the SaI cells with formalin generally yields results comparable to those found with mitomycin treatment (data not shown).

Table I summarizes an experiment designed to look at the effect of 0.15 Gy upon T cell subsets in mice immunized with small numbers of SaI cells. On day 0, mice were exposed to 0.15 Gy or sham irradiated and then injected with 150 SaI cells. A control group was injected with PBS. On day 5, mice from each group were sacrificed for an assessment of lymphocyte subsets. With respect to the relative proportion of T cell subsets in lymph node, injection of mitomycin-treated SaI cells (Group II) is associated with a marked decrement in the Lyt-$1^+2^+$ phenotype with corresponding increments in the other two T cell subsets, particularly the Lyt-$1^-2^+$ phenotype. Irradiation administered prior to immunization appears to mute this shift, especially with respect to the Lyt-$1^-2^+$ phenotype. Corresponding observations with spleen cells show no consistent differences among the groups.

The basis for the progressive growth of tumours known to be antigenic to the autochthonous host is not well understood. A variety of mechanisms have been proposed to explain this apparent paradox including antigenic modulation (Stackpole & Jacobson, 1978) and blocking factors (Hellstrom & Hellstrom, 1977). Recently, suppressor T cells have

**Figure 3** Effects of 0.15 Gy upon response of A/J mice to lethally irradiated SaI cells.*
*Experimental approach similar to that described in legend for **Figure 1** except that tumour cells were inactivated by exposure to 50 Gy.

**Table I** Distribution by Lyt phenotype of splenic and lymph node T cells in mice exposed to 0.15 Gy and injected with subimmunogenic numbers of mitomycin-treated SaI cells[a]

| *Group* | *Radiation dose* | *No. mitomycin-treated SaI cells* | *Tissue assessed* | *T cell subsets* $Lyt\text{-}1^+2^+$ | $Lyt\text{-}1^+2^-$ | $Lyt\text{-}1^-2^+$ | *Other* |
|---|---|---|---|---|---|---|---|
| I | 0 Gy | — | Spleen | 12 | 21 | 4 | 63 |
| | | | Lymph node | 21 | 39 | 6 | 34 |
| II | 0 Gy | 150 | Spleen | 12 | 22 | 2 | 64 |
| | | | Lymph node | 0 | 48 | 18 | 34 |
| III | 0.15 Gy | 150 | Spleen | 5 | 26 | 10 | 59 |
| | | | Lymph node | 8 | 49 | 7 | 36 |

[a]Groups of 30 mice were exposed to 0.15 Gy or sham irradiated and then immunized with 150 mitomycin-treated SaI cells. A control group was injected with PBS. On day 5 after irradiation and injection, 5 mice from each group were sacrificed for a determination of T cell subsets in spleen and lymph node. The remainder of the mice were injected with $10^4$ SaI cells on day 21 and followed for tumour size.

also been implicated (Schatten *et al.*, 1984; Spellman & Roberts, 1983; Carter *et al.*, 1983). Whatever the mechanism, however, the end result is that the host appears to be tolerant, or partially tolerant, to the tumour.

Whole body irradiation has been employed both to terminate and to generate tolerance. The involved doses, however, have been in the low to mid-lethal dose range (Nossal & Larkin, 1959; Anderson & Warner, 1976) and thus considerably greater than those employed herein.

In the present series of experiments, whole body exposure to 0.15 Gy prior to immunization results in partial immunity in both the 'tolerant' and the 'nonimmune' groups. This observation suggests that the absence of a demonstrable anti-tumour response in the sham irradiated animals exposed to $10^2$ mitomycin-treated cells (Figure 2) does not indicate that the number of cells is below the threshold required to initiate a response. Rather, it implies that $10^2$ SaI cells trigger a balanced state of immunity between the effector and suppressor components. The consequence is no observable deviation from the control situation. Low dose irradiation, which inhibits the tumour-associated shifts in T cell subsets, appears to perturb this balance and permits the anti-tumour effector component to predominate. A somewhat analogous situation has been reported in rats (Baldwin *et al.*, 1982) and mice (Perry & Greene, 1982) treated with low dose cyclophosphamide prior to treatment with a KCl extraction of rat hepatoma or heavily irradiated S1509 plus anti-I-$A^k$ alloantiserum respectively; these two priming regions were ineffective in the absence of cyclophosphamide. Preliminary experiments suggest that low doses of cyclophosphamide and radiation, used conjointly, are more effective in permitting immunization with small numbers of mitomycin-treated SaI cells than in either agent employed alone. Spleen cells from mice immunized in this fashion can be employed to adoptively transfer partial immunity to adult thymectomized-lethally irradiated-bone marrow restored recipients.

Inactivation of tumour cells with formalin but not lethal (50 Gy) irradiation yields results comparable to those with mitomycin. The basis of this difference among inactivating agents is not known but may relate to the marked alterations caused by irradiation to the surface topography of susceptible cells (Anderson & Warner, 1976).

Supported by the Veterans Administration

**References**

ANDERSON, R.E., TOKUDA, S., WILLIAMS, W.L. & WARNER, N.L. (1982). Radiation-induced augmentation of the response of A/J mice to SaI tumor cells. *Am. J. Pathol.*, **108**, 24.

ANDERSON, R.E. & WARNER, N.L. (1976). Ionizing radiation and the immune response. *Adv. Immunol.*, **24**, 215.

BALDWIN, R.W., BYERS, V.S., HANNANT, D., JONES, J.A., PIMM, M.V. & PRICE, M.R. (1982). Cellular interactions modulating host resistance to tumours. In *Recent Results in Cancer Research*, **80**. Mathe *et al.* (eds) p. 338, Springer-Verlag, Berlin.

CANTOR, H. & GERSHON, R.K. (1979). Immunological circuits: Cellular composition. *Fed. Proc.*, **38**, 2058.

CARTER, R.H., DREBIN, J.A., SCHATTEN, S., PERRY, L.L. & GREENE, M.I. (1983). Regulation of the immune responses to tumor antigens. IX. *In vitro* Lyt-$1^+2^-$ cell proliferative responses to cellbound or subcellular tumor antigen. *J. Immunol.*, **130**, 997.

GERSHON, R.K. (1980). Immunoregulation circa 1980: Some comments on the state of the art. *J. Allergy Clin. Immunol.*, **66**, 18.

HELLSTROM, K.E. & HELLSTROM, I. (1977). Immunologic enhancement of tumor growth. In *Mechanisms of Tumor Immunity*. Greene *et al.* (eds) p. 147. John Wiley & Sons, New York.

LANIER, L.L., WARNER, N.L., LEDBETTER, J.A. & HERZENBERG, L.A. (1981). Quantitative immunofluorescent analysis of surface phenotypes of murine B cell lymphomas and plasmacytomas with monoclonal antibodies. *J. Immunol.*, **127**, 1691.

NOSSAL, G.J.V. & LARKIN, L. (1959). Breakdown of immunological tolerance following irradiation. *Aust. J. Sci.*, **22**, 168.

NOSSAL, G.J.V., PIKE, B.L., STOCKER, J.W., LAYTON, J.E. & GODING, J.W. (1976). Hapten-specific B lymphocytes: Enrichment, cloning, receptor analysis, and tolerance induction. *Cold Spring Harbor Symp. Quant. Biol.*, **41**, 237.

PERRY, L.L. & GREENE, M.I. (1982). Conversion of immunity to suppression by *in vivo* administration of I-A subregion-specific antibodies. *J. Exp. Med.*, **156**, 480.

SCHATTEN, S., GRANSTEIN, R.D., DREBIN, J.A. & GREENE, M.I. (1984). Suppressor T cells and the immune response to tumors. *CRC Critical Reviews in Immunology*, **4**, 335.

SOKAL, R.R. & ROHLF, F.J. (1969). *Biometry: The Principles and Practice of Statistics in Biological Research*. W.H. Freeman and Co., San Francisco.

SPELLMAN, C.W. & ROBERTS, L.K. (1983). Induction of suppressor T cells following UVL exposure of animals and their role in the regulation of immune responses to developing tumors. In *Experimental and Clinical Photoimmunology.*, **2**, Daynes & Krueger (eds) p. 7. CRC Press, Inc., Boca Raton.

STACKPOLE, C.W. & JACOBSON, J.B. (1978). Antigenic modulation. In: *The Handbook of Cancer Immunology. Cellular Escape from Immune Destruction.* **2**, Waters (ed) p. 55. Garland STPM Press, New York.